# Development and Evaluation of Liquid and Solid Self-Emulsifying Drug Delivery Systems for Atorvastatin

**DOI:** 10.3390/molecules201219745

**Published:** 2015-11-25

**Authors:** Anna Czajkowska-Kośnik, Marta Szekalska, Aleksandra Amelian, Emilia Szymańska, Katarzyna Winnicka

**Affiliations:** 1 Department of Pharmaceutical Technology, Medical University of Białystok, Mickiewicza 2c, 15-222 Białystok, Poland; marta.przybyslawska@umb.edu.pl (M.S.); aleksandra.amelian@umb.edu.pl (A.A.); esz@umb.edu.pl (E.S.); 2 Department of Clinical Pharmacy, Medical University of Białystok, Mickiewicza 2d, 15-222 Białystok, Poland

**Keywords:** atorvastatin, self-emulsifying drug delivery system (SEDDS), spray drying technique, lipid based formulation, poorly water soluble drug

## Abstract

The objective of this work was to design and characterize liquid and solid self-emulsifying drug delivery systems (SEDDS) for poorly soluble atorvastatin. To optimize the composition of liquid atorvastatin-SEDDS, solubility tests, pseudoternary phase diagrams, emulsification studies and other *in vitro* examinations (thermodynamic stability, droplet size and zeta potential analysis) were performed. Due to the disadvantages of liquid SEDDS (few choices for dosage forms, low stability and portability during the manufacturing process), attempts were also made to obtain solid SEDDS. Solid SEDDS were successfully obtained using the spray drying technique from two optimized liquid formulations, CF3 and OF2. Despite liquid SEDDS formulation, CF3 was characterized by lower turbidity, higher percentage transmittance and better self-emulsifying properties, and based on the *in vitro* dissolution study it can be concluded that better solubilization properties were exhibited by solid formulation OF2. Overall, the studies demonstrated the possibility of formulating liquid and solid SEEDS as promising carriers of atorvastatin. SEDDS, with their unique solubilization properties, provide the opportunity to deliver lipophilic drugs to the gastrointestinal tract in a solubilized state, avoiding dissolution—a restricting factor in absorption rate of BCS Class 2 drugs, including atorvastatin.

## 1. Introduction

The majority of new drugs exhibit poor aqueous solubility, which affects their low bioavailability after oral delivery. Many strategies have been described to increase the dissolution rate of drugs by reducing their particle size and salt formation, using surfactants, cyclodextrins, liposomes or nanoparticles [1,2,3,4]. A relatively new approach for poorly soluble drugs is lipid-based formulations, particularly self-emulsifying drug delivery systems (SEDDS) [5,6].

SEDDS are isotropic mixtures of oils and surfactants with or without co-surfactants, which act as lipid-based formulations after oral application in aqueous gastrointestinal fluid and upon gentle agitation can form an oil-in-water emulsion [7,8,9,10]. SEDDS technology was employed to increase solubility and consequently the bioavailability of many poorly water soluble drugs such as phyllanthin, celastrol, ketoprofen, indomethacin and hydrocortisone [11,12,13,14].

SEDDS as liquid formulations have several disadvantages such as low drug loading capacity, drug leakage, low stability, and few choices of dosage forms. To overcome these limitations, liquid SEDDS (L-SEDDS) can be transformed to solid dosage forms by using different methods (filling capsules with liquid or semi-solid SEDDS, adsorption to solid carrier, melt granulation, spray drying, melt extrusion or nanoparticle formation) [15,16]. Solid self-emulsifying drug delivery systems (S-SEDDS) combine the advantages of liquid lipid formulations with those of solid dosage forms such as higher stability and longer period of storage [17,18]. S-SEDDS could be formulated in the form of self-emulsifying capsules, pellets/tablets, micro/nano-particles, suppositories or dry emulsions. One of the methods used for the conversion of L-SEDDS to S-SEDDS is spray drying. This technique allows preparation of the self-emulsifying dry emulsion by removing water from an emulsion containing a water-soluble solid carrier [19,20,21].

Atorvastatin (ATR) inhibits 3-hydroxy-3-methylglutaryl coenzyme A (HMG-CoA) reductase, an enzyme found in liver tissue that plays a key role in cholesterol production [22]. ATR is commonly used in hyperlipidemia and cardiovascular events. It is insoluble in pH < 4 and very slightly soluble in water and in pH 7.4 phosphate buffer (<1 mg/mL). Low oral bioavailability of ATR (only 12% after a 40 mg oral dose) is associated with its poor solubility in water and high (above 80%) presystemic clearance [23,24,25]. Therefore, using different lipid carriers of ATR has been widely reported as a promising drug delivery system [26,27,28,29,30]. As to our best knowledge there are no papers concerning solid SEDDS for ATR, the aim of this work was to design and to obtain both liquid and solid SEDDS from the same, optimized composition for solubility enhancement of ATR. To screen and to optimize the composition of L-SEDDS, solubility tests, pseudoternary phase diagrams, self-emulsifying grading tests, determination of percentage transmittance, refractive index and turbidity, droplet size and zeta potential analysis were performed. The optimized L-SEDDS were finally converted to S-SEDDS using the spray drying technique and characterized.

## 2. Results and Discussion

### 2.1. Solubility of Atorvastatin (ATR)

The high solubility of drugs in the oil phase is crucial parameter in designing stable SEDDS formulations. The drug should possess good solubility in solvent, so precipitation during the shelf life of the formulation and after dilution in water phase can be avoided [3,31]. The solubility of ATR in selected oils, surfactants and co-surfactants is presented in Table 1. The maximum solubility of ATR was observed in oils such as Capryol 90 (14.91 mg/mL) and oleic acid (6.50 mg/mL), surfactants such as Labrasol (33.31 mg/mL), Caprol MPGO (29.83 mg/mL), Kolliphor RH40 (29.67 mg/mL) and Tween 80 (18.53 mg/mL), and co-surfactants as 1,2-propylene glycol (>1 g/mL) and PEG 400 (34.04 mg/mL). The components that provided the best solubility of ATR were further used to develop the pseudoternary phase diagrams. Surfactants can cause gastrointestinal irritation, so their selection is a very important factor in SEDDS design. The non-ionic surfactants are less toxic than ionic ones and they are characterized by lower critical micelle concentration values [2]. Another important criterion is selection of surfactant with proper hydrophilic-lipophilic balance (HLB) value. Generally, to create SEDDS, surfactants with HLB values 12–15 are recommended [32]. The selected surfactants, Caprol MPGO, Kolliphor RH40, Labrasol and Tween 80, have HLB values of 8.5, 14.0–16.0, 14.0 and 15.0, respectively [33].

**Table 1:** Solubility of ATR in (**A**) oils; (**B**) surfactants and (**C**) co-surfactants.

Solvent	Solubility (mg/mL)	Solvent	Solubility (mg/mL)
(**A**) Oil		Caprol PGE-860	13.25 ± 0.48
Almond oil	2.82 ± 0.11	Kolliphor RH40	29.67 ± 0.30
Capryol 90	14.91 ± 0.34	Labrafil M19944CS	2.80 ± 0.06
Captex 200P	0.23 ± 0.01	Labrasol	33.31 ± 0.43
Castor oil	2.32 ± 0.10	Lauroglycol FCC	1.85 ± 0.06
Linseed oil	0.29 ± 0.01	Span 20	14.69 ± 0.28
Miglyol 812	0.89 ± 0.05	Tween 20	13.28 ± 0.08
Oleic acid	6.50 ± 0.21	Tween 60	17.91 ± 0.63
Rapeseed oil	1.32 ± 0.07	Tween 80	18.53 ± 0.46
Soja oil	1.05 ± 0.04	(**C**) Co-surfactant	
(**B**) Surfactant		1,2-propylene glycol	>1 g
Caprol MPGO	29.83 ± 0.87	PEG 400	34.04 ± 1.21

### 2.2. Pseudoternary Phase Diagrams

Self-emulsifying drug delivery systems upon their introduction into aqueous media form oil-in-water emulsions with only gentle agitation. The surfactant and co-surfactant adsorb at the interface and reduce the interfacial energy. The decrease in the free energy required for the emulsion formation improves the thermodynamic stability of formulation [3,34]. Therefore, the selection of oil and surfactant plays an important role in the SEDDS designing.

**Figure 1:**
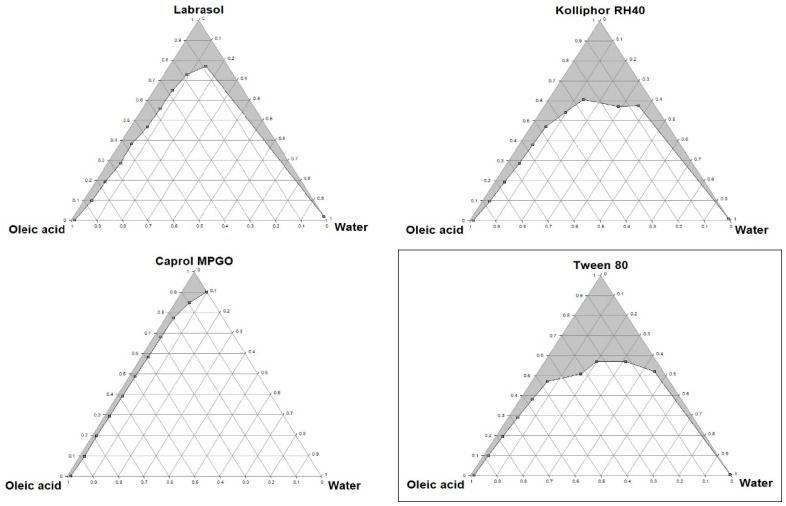

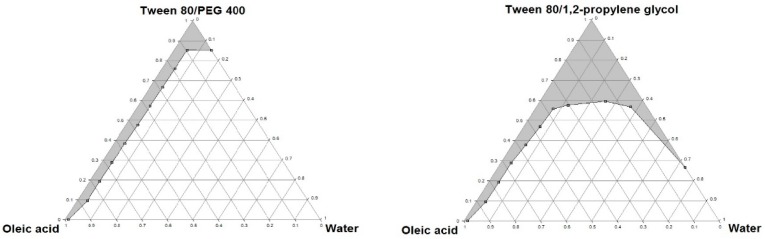
Pseudoternary phase diagrams of mixtures with oleic acid. Tween 80/PEG 400 and Tween 80/1,2-propylene glycol ratio is 1:1. The diagram with the formulation characterized by the best self-emulsifying properties is framed.

**Figure 2:**
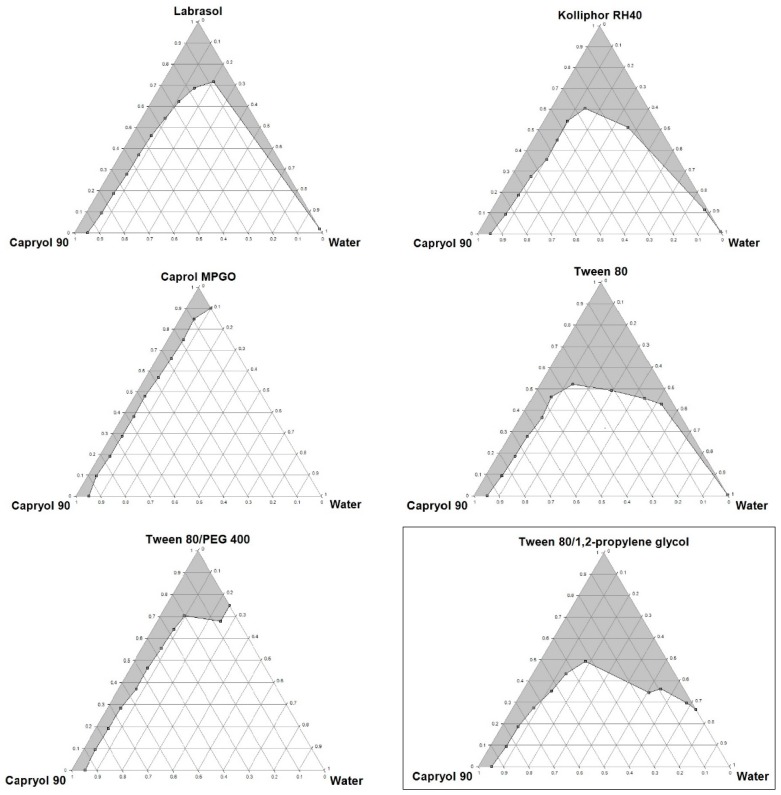
Pseudoternary phase diagrams of mixtures with Capryol 90. Tween 80/PEG 400 and Tween 80/1,2-propylene glycol ratio is 1:1. The diagram with the formulation characterized by the best self-emulsifying properties is framed.

Pseudoternary phase diagrams were constructed to identify the self-emulsifying regions and to determine the optimum concentrations of oil, surfactant and co-surfactant. With the increase in the surfactant concentration, an increase in the self-emulsifying region was observed. The phase diagrams of oil-surfactant-water systems in different ratios are shown in Figure 1 and Figure 2. The pseudoternary phase diagrams were constructed to optimize the concentration of the oleic acid and Capryol 90 (oils), Caprol MPGO, Kolliphor RH40, Labrasol and Tween 80 (surfactants), PEG 400 and 1,2-propylene glycol (co-surfactants) and to identify their effect on emulsion formation. The stable formulation region in the pseudoternary phase diagram was marked in a gray colour.

Based on the pseudoternary phase diagrams, the formulations with the best self-emulsifying properties, containing oleic acid (5%–50%) with Tween 80 (50%–95%), and Capryol 90 (5%–50%) with Tween 80/1,2-propylene glycol 1:1 (50%–95%), were selected for further studies.

Based on the results obtained from the pseudoternary phase diagrams, the formulations with the optimal self-emulsifying properties were prepared. L-SEDDS were formulated with varying ratios of oil, surfactant and co-surfactant as shown in Table 2.

**Table 2:** Composition of liquid atorvastatin-SEDDS. In all the formulations the amount of atorvastatin was constant (10 mg).

Formulation Code	Oleic Acid (%)	Tween 80 (%)	Formulation Code	Capryol 90 (%)	Tween 80/1,2-Propylene Glycol (1:1) (%)
OF1 *	5	95	CF1 **	5	95
OF2 *	10	90	CF2 **	10	90
OF3 *	15	85	CF3 **	15	85
OF4 *	20	80	CF4 **	20	80
OF5 *	25	75	CF5 **	25	75
OF6 *	30	70	CF6 **	30	70
OF7 *	35	65	CF7 **	35	65
OF8 *	40	60	CF8 **	40	60
OF9 *	45	55	CF9 **	45	55
OF10 *	50	50	CF10 **	50	50

* formulations OF1-OF10 were prepared using oleic acid as oil and Tween 80 as surfactant; ** formulations CF1-CF10 were prepared with Capryol 90 as oil, Tween 80 as surfactant and 1,2-propylene glycol as co-surfactant.

### 2.3. Thermodynamic Stability and Phase Separation Studies

SEDDS should be stable in different temperature conditions and not lose their spontaneous emulsification ability upon dilution [16,35]. Therefore, to observe the capacity of designed SEDDS to withstand stress conditions, thermodynamic stability evaluation and phase separation studies were performed. All formulations were submitted to the stressed thermodynamic conditions by using centrifugation, freezing and thawing cycle tests. It was observed that all prepared SEDDS survived the thermodynamic stability tests and no sign of phase separation was observed.

Phase separation studies after 100 times dilution with water revealed that formulations OF1–OF6 and CF1–CF6 were stable for a period of 24 h, so these formulations were used for further studies.

### 2.4. Emulsification Study

Emulsification studies are an important method to evaluate the self-emulsifying properties of designed formulations. When subjected to aqueous dilution under mild agitation, SEDDS should completely and rapidly disperse. Surfactants present in the SEDDS reduce the interfacial tension between oil and aqueous phases and facilitate dispersion and formation of oil-in-water emulsion [26]. The results of the emulsification study are presented in Table 3. Formulations CF1–CF2 were assessed as Grade A (within 1 min forming clear and bluish microemulsion). Formulations CF3–CF4 exhibited Grade B (within 1 min forming slightly less clear emulsion with a bluish white appearance) and CF5–CF6–Grade C (within 2 min forming fine milky emulsion). SEDDS containing oleic acid demonstrated Grade C/A for formulation OF1 and Grade D/C for formulations OF2–OF6 (Grade D—longer than 2 min forming greyish white emulsion having slightly oil appearance) [7]. Due to the differences in appearance of the emulsion and the time of its forming, it was difficult to determine their one specific degree.

**Table 3:** Emulsification study observation of formulations OF1–OF6 and CF1–CF6 (grade and emulsification time).

Formulation	Grade **	Emulsification Time (s)	Formulation	Grade **	Emulsification Time (s)
OF1	C */A	75	CF1	A	15
OF2	D */C	200	CF2	A	15
OF3	D */C	356	CF3	B	20
OF4	D */C	420	CF4	B	20
OF5	D */C	458	CF5	C	65
OF6	D */C	480	CF6	C	65

* refers to emulsification time; ** characteristics of grades is presented in the Experimental Section (Subsubsection 3.6.2.).

### 2.5. Determination of Percentage Transmittance, Refractive Index and Turbidity

If refractive index is similar to the refractive index of water (1.333) and percentage transmittance above 90%, then formulations have a transparent nature [7]. The results of percentage transmittance, refractive index and turbidity studies are shown in Table 4. Formulations OF1 and CF1–CF3 have transmittance value greater than 90%, suggesting their clarity. This might be due to the smaller particle size, which increases the transparency of the emulsion. The refractive index of all formulations was similar to the refractive index of water. The turbidity of formulations containing Capryol 90 (CF1–CF6) was lesser than the turbidity of formulations with oleic acid as oily phase (OF1–OF6). The OF1 and CF1–CF4 formulations had turbidity below 100 NTU, which confirms good (OF1, CF1–CF2) or relatively good (CF3–CF4) clarity.

**Table 4:** Percentage transmittance (%T), refractive index (n_D_) and turbidity (NTU) of formulations OF1–OF6 and CF1–CF6.

Formulation	%T	n_D_	NTU *	Formulation	%T	n_D_	NTU *
OF1	99.7	1.334	12.8	CF1	99.9	1.334	0.362
OF2	67.1	1.334	104	CF2	98.8	1.334	0.452
OF3	54.4	1.334	302	CF3	93.4	1.334	32.5
OF4	48.5	1.334	473	CF4	77.6	1.334	77.6
OF5	35.0	1.334	588	CF5	35.6	1.334	222
OF6	23.1	1.334	476	CF6	3.0	1.334	591

* Nephelometric Turbidity Unit.

### 2.6. Droplet Size and Zeta Potential Determination

The droplet size of the emulsion determines the absorption and bioavailability of the drug—smaller droplets provide a larger surface area, leading to faster drug release into aqueous medium [2]. The effect of the emulsion droplet size on the permeation for intestinal mucosa has been investigated by Gershanik *et al.* [36]. They found that the optimal droplet size was in the range of 100–500 nm.

The results of droplet size and zeta potential determinations are shown in Table 5. It was found that OF1–OF2 and CF1–CF3 formulations were characterized by a mean droplet size below 200 nm. The increase in the particle size observed in formulations OF3–OF6 and CF4–CF6 was probably due to the increase in the ratio of the oil phase and simultaneous decrease in the surfactant amount.

The zeta potential is used to identify the charge of the droplets. The value of zeta potential indicates the degree of electrostatic repulsion between particles in a dispersion. The high zeta potential provides stability of dispersion and prevents aggregation. The SEDDS formulations with zeta potential greater than ±30 mV are characterized as stable. In conventional SEDDS, the charge of an oil droplet is negative because of the presence of free fatty acids [20,37]. The SEDDS formulations OF2–OF6 and CF3–CF6 showed optimal values of zeta potential (−32.0 to −39.7 mV and −30.2 to −36.6 mV, respectively), which indicated their stability. It was also observed that surfactant addition decreasing the particle size led to an increase in the zeta potential value.

**Table 5:** Particle size and zeta potential of formulations OF1–OF6 and CF1–CF6.

Formulation	Particle Size (nm)	Zeta Potential (mV)	Formulation	Particle Size (nm)	Zeta Potential (mV)
OF1	65.16 ± 1.22	−24.8 ± 1.81	CF1	14.56 ± 0.13	−2.98 ± 0.43
OF2	190.5 ± 1.28	−32.0 ± 3.20	CF2	76.22 ± 1.19	−5.39 ± 0.22
OF3	224.8 ± 1.36	−32.8 ± 2.81	CF3	188.1 ± 1.48	−30.2 ± 1.21
OF4	393.6 ± 2.38	−33.3 ± 3.02	CF4	267.3 ± 0.56	−31.0 ± 1.01
OF5	412.6 ± 2.42	−37.3 ± 2.76	CF5	297.1 ± 2.53	−34.6 ± 1.77
OF6	447.4 ± 1.52	−39.7 ± 3.37	CF6	319.6 ± 2.42	−36.6 ± 2.32

### 2.7. Preparation of S-SEDDS Formulations

Due to the fact that L-SEDDS possess some disadvantages [15,16], an effort to obtain solid SEDDS was made. Based on the obtained results, optimal L-SEDDS formulations (OF2 and CF3) were used to prepare S-SEDDS using the spray-drying technique. In this method, liquid formulation (containing drug, oil, surfactant, solid carriers and water) is atomized into a spray of droplets, which are introduced into a drying chamber, where the water evaporates, forming dry particles referred to as solid SEDDS. S-SEDDS spontaneously form emulsions in aqueous solution and solve the stability problems of classic emulsion during storage (phase separation, contamination by microorganism). Moreover, they can be further formed into solid dosage forms, as multicompartment tablets or capsules [19].

S-SEDDS were successfully prepared by the spray drying technique, and ATR content in examined formulations is presented in Table 6.

**Table 6:** Atorvastatin (ATR) content in liquid and solid SEDDS.

Formulation	Drug Content (the Amount of ATR per 1 g of Formulation)	Encapsulation Efficiency (%)
L-SEDDS OF2	9.46 mg	-
L-SEDDS CF3	21.78 mg	-
S-SEDDS OF2	4.64 mg	52.9
S-SEDDS CF3	11.17 mg	53.6

**Figure 3:**
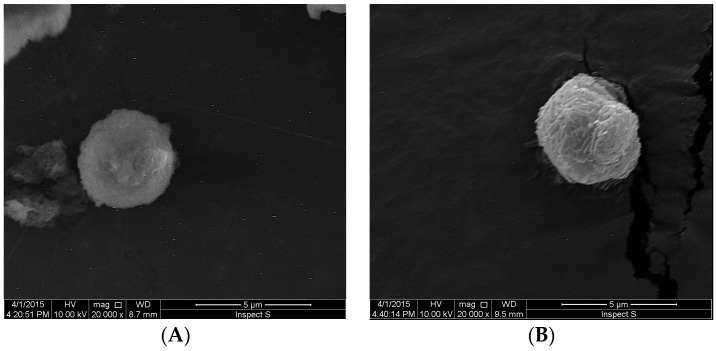
Representative SEM images of S-SEDDS microparticles: (**A**) formulation OF2; (**B**) formulation CF3 (magnification 20,000×).

SEM images of obtained S-SEDDS containing ATR are shown in Figure 3. The SEM photographs of S-SEDDS showed that the obtained particles had a size about 4 μm and were spherical in shape. However, the particles of OF2 formulation were characterized by a more smooth surface.

### 2.8. In Vitro Dissolution Study

**Figure 4:**
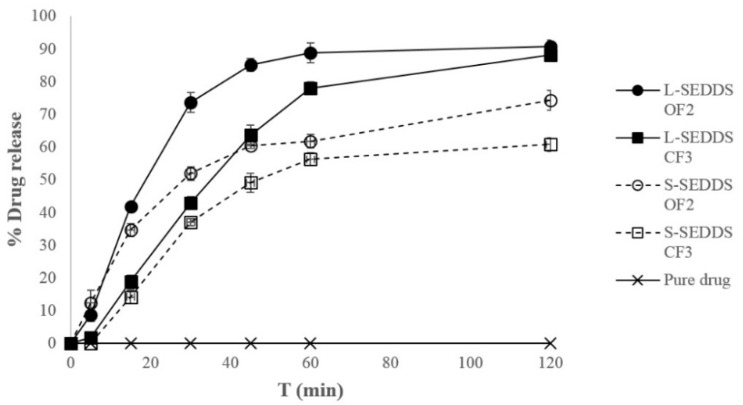
*In vitro* dissolution profile of ATR from optimized liquid- (L) and solid- (S) SEDDS formulations.

The release behaviour of ATR from the optimized liquid and solid SEDDS is presented in Figure 4. Drug release was significantly increased in formulated SEDDS as compared to pure ATR powder (*p* < 0.05). Interestingly, for the first 15 min of the study, no significant differences between liquid and solid SEDDS were noted. This might be due to the quick emulsification properties of all analyzed SEDDS and their ability to keep drugs in a solubilized state upon dilution, which resulted in significantly a greater rate of ATR release into the aqueous medium. However, ATR release from liquid SEDDS after 30 min was higher than from solid SEDDS formulations. The percentages of cumulative ATR release after 30 min from liquid and solid formulations OF2 and CF3 containing lactose as a carrier were about 72%, 51%, 42%, and 36%, respectively. The droplet size of microemulsion formed after adding water is an important factor influencing the rate of dissolution into aqueous phase. The liquid OF2, CF3 and solid OF2 SEDDS formed microemulsions with smaller droplet size (below 200 nm) and further improved the ATR dissolution compared to solid SEDDS formulation CF3 with about 330 nm droplet size. SEDDS, with their unique solubilization properties, offer the opportunity to deliver lipophilic drugs to the gastrointestinal tract in a dissolved state, avoiding the dissolution step, which is a restricting factor in absorption rate of BCS Class 2 drugs, including ATR.

## 3. Experimental Section

### 3.1. Chemicals

Atorvastatin (ATR) was obtained as a gift from Biofarm (Poznań, Poland). Captex 200P and Caprol PGE-860 were gift samples from Abitec Corporation (Janesville, WI, USA). Capryol 90, Caprol MPGO, Lauroglycol FCC, Labrafil M19944CS and Labrasol were obtained from Gattefosse (Nanterre, France). Orthophosphoric acid, oleic acid, Span 20, Kolliphor^®^RH40, Tween 20, Tween 60, Tween 80 and polyoxyethylene glycol 400 (PEG 400) were purchased from Sigma Aldrich (Saint Louis, MO, USA). Rapeseed oil and Castor oil were obtained from Fagron (Kraków, Poland). Soja oil and Miglyol 812 were purchased from Caesar & Loretz GmbH (Hilden, Germany). Almond oil was obtained from Chempol (Wrocław, Poland). Linseed oil was purchased from Amara (Kraków, Poland). 1,2-propylene glycol was obtained from POCH (Gliwice, Poland). Potassium dihydrogen phosphate was obtained from Chempur (Piekary Śląskie, Poland). Lactochem^®^ powder (lactose) was a gift from DFE pharma (Goch, Germany). HPLC-grade water was prepared by a Milli-Q Reagent Water System (Millipore, Billerica, MA, USA). Acetonitrile and methanol (Merck, Darmstadt, Germany) used in the present study were of HPLC-grade. All other reagents were of analytical grade.

### 3.2. Solubility Studies

The study was carried out by taking 1.0 mL of various solvents (oils, surfactants and co-surfactants) in a capped vial containing an excess amount (100 mg) of ATR. The mixtures were vortexed for 1 min to facilitate uniform dispersion, then were agitated with shaker for 30 min at 40 °C, and next for 48 h at room temperature. Afterwards, the samples were centrifuged at 3000 rpm for 15 min. The supernatant was collected and diluted with methanol. The concentration of dissolved ATR was determined by the HPLC method.

### 3.3. HPLC Analysis

The concentration of ATR was determined by the high-performance liquid chromatographic (HPLC) method. The system consists of Agilent Technologies 1260 Infinity with a UV detector (Agilent, Waldbronn, Germany). Isocratic separation was achieved on a Waters Spherisorb^®^ ODS, 4.6 mm × 250 mm, 5 μm column (Waters Corporation, Milford, MA, USA). Mobile phase was acetonitrile/potassium dihydrogen phosphate buffer pH 3.0 (60:40; *v*/*v*), the flow rate was 1.0 mL/min and UV detection was performed at a wavelength of 247 nm. The column temperature was maintained at 25 °C. For the injection into the HPLC system, 20 μL of sample was used. The retention time of ATR was 5.03 min. Standard calibration curve was linear over the range of 5–100 μg/mL with the correlation coefficient (R^2^) 0.999.

### 3.4. Construction of Ternary Phase Diagrams

Ternary phase diagrams were constructed for mixtures of oil, surfactant/co-surfactant and water at room temperature, using the water titration method. The mixtures of oil and surfactant/co-surfactant at certain weight ratios were diluted with water in a dropwise manner. For each phase diagram, oil and surfactant were mixed thoroughly in different weight ratios from 1:9 to 9:1 (1:9, 2:8, 3:7, 4:6, 5:5, 6:4, 7:3, 8:2, 9:1) in different glass vials. The homogenous mixture of oil and surfactant/co-surfactant was formed by vortexing for 5 min. Then each mixture was titrated with water and visually observed for phase clarity and flowability. The amount of water at which turbidity-to-transparency and transparency-to-turbidity transitions occurred was derived from the weight measurements. These values were then used to determine the boundaries of the microemulsion area corresponding to the values of oil and surfactant/co-surfactant. The phase diagrams were constructed using ProSim Ternary Diagram software (ProSim SA, Labege, France).

### 3.5. Preparation of Liquid SEDDS

L-SEDDS (Table 2) were prepared by dissolving ATR in selected mixtures of oil and surfactant/co-surfactant. The amount of formulation should solubilize the whole drug dose (for ATR single dose is 10 mg). Hence, based on the solubility studies, 10 mg of ATR was dissolved in 1 g of formulations with oleic acid (OF1–OF10) and in 0.5 g of formulations with Capryol 90 (CF1–CF10). The mixtures were shaken and heated at 40 °C for a time necessary to dissolve the drug completely.

### 3.6. Characterization of Liquid SEDDS

#### 3.6.1. Thermodynamic Stability and Phase Separation Study

SEDDS formulations were subjected to 3 freeze-thaw cycles, which included freezing at −18 °C for 24 h followed by thawing at 40 °C for 24 h. After centrifugation at 3000 rpm for 15 min, the formulations were observed for phase separation. Only stable formulations were selected for further experiments.

Phase separation study was assessed by exposing SEDDS formulations to 100 fold dilution with distilled water. Examined formulations were stored at 25 °C for 24 h and observed visually for phase separation and precipitation of drug.

#### 3.6.2. Emulsification Study

The emulsification study was performed in a USP dissolution tester (DT 600 HH, Erweka, Heusenstamm, Germany). Each formulation (1 mL) was added to 100 mL distilled water maintained at 37 °C, with paddle rotating at 100 rpm for gentle agitation. The *in vitro* performance of designed formulations was visually assessed by using the grading system as shown below [7]:

**Grade A:**  Rapidly forming (within 1 min) emulsion, with a clear or bluish appearance
**Grade B:**  Rapidly forming (within 1 min), slightly less clear emulsion, with a bluish white appearance
**Grade C:**  Fine milky emulsion that formed within 2 min
**Grade D:**  Dull, greyish white emulsion having slightly oily appearance that is slow to emulsify (longer than 2 min)
**Grade E:**  Formulation exhibiting either poor or minimal emulsification with large oil droplets on the surface

#### 3.6.3. Determination of Percentage Transmittance, Refractive Index and Turbidity

The SEDDS formulations were diluted 100 times with water. The percentage of transmittance of the prepared emulsions was measured using UV spectrophotometer (Hitachi, Tokyo, Japan) keeping distilled water as blank at 630 nm. The refractive index was measured using Abbe’s refractometer (Atago, Tokyo, Japan). Turbidity of all formulations was studied using turbidimeter (Hach Lange, Düsseldorf, Germany), results were given as nephelometric turbidity unit (NTU).

#### 3.6.4. Droplet Size and Zeta Potential Analysis

The particle size and zeta potential of obtained emulsions (after dilution 100 times with water) was determined by using Zetasizer Nano ZS90 (Malvern Instruments, Malvern, UK).

#### 3.6.5. Drug Content in L-SEDDS

An amount of SEDDS equivalent to 10 mg of ATR was carefully weighted and placed in 100 mL volumetric flask containing methanol. After agitating for 24 h in a water bath (250 rpm), the extracted solution was analyzed for ATR content as described in Subsection 3.3.

### 3.7. Preparation of Solid SEDDS

The S-SEDDS were prepared using the spray-drying technique using aBüchi Mini Spray Dryer B-290 apparatus (Büchi, Flawil, Switzerland). 10 g of lactose (Lactochem^®^ powder) was dissolved in 100 mL distilled water and then the liquid SEDDS (10 g) was added. The solution was kept at 50 °C for 15 min to obtain an emulsion. The emulsion was spray dried under the conditions set during preliminary experiments: inlet temperature 60 °C; outlet temperature 40 °C; aspiration 100%, spray flow 550 L/h; feeding rate of the emulsion 4 mL/min.

### 3.8. Characterization of Solid SEDDS

#### 3.8.1. Drug Content in S-SEDDS

ATR loading in S-SEDDS was determined by dissolving accurately weighted amount of dried emulsion (50 mg) in 10 mL of methanol and agitating for 24 h at 250 rpm in a water bath. After filtration using a 0.45 μm cellulose acetate membrane filter (Chromafil^®^, Düren, Germany), the concentration of ATR was determined as described in Subsection 3.3. ATR encapsulation efficiency (EE %) in S-SEDDS was calculated by the formula: EE = Q_a_/Q_t_ × 100, where: EE—percentage of encapsulation efficiency, Q_a_—actual drug content, Q_t_—theoretical drug content [38].

#### 3.8.2. Morphological Analysis

The morphological features of solid atorvastatin-SEDDS were assessed by scanning electron microscope (SEM) (Hitachi S4200, Tokyo, Japan). Before imaging samples were sputter-coated with gold.

#### 3.8.3. Droplet Size Determination

Droplet size of emulsions obtained from solid SEDDS was determined by using Zetasizer Nano ZS90 as described in Subsubsection 3.6.4.

### 3.9. In Vitro Dissolution Study

For the *in vitro* dissolution study USP apparatus type I (Erweka Dissolution tester type DT 600HH, Heusenstamm, Germany) was used. Selected liquid and solid SEDDS formulations equivalent to 10 mg of ATR and only 10 mg of ATR powder were filled in hard gelatin capsules (size 00) and were undertaken for dissolution study. 300 mL of water maintained at 37 °C and stirred at 100 rpm was used as dissolution medium. Dissolution samples (5 mL) were withdrawn at predetermined time intervals and replaced with an equivalent amount of fresh water. Samples were filtered through a 0.45 μm nylon membrane filter (Chromafil^®^) and concentrations of ATR were determined by HPLC method as described in Subsection 3.3.

### 3.10. Statistical Analysis

Results are expressed as the mean and standard deviation. The data were statistically analyzed using the Mann-Whitney test (Statistica 10.0 software, StatSoft, Tulsa, OK, USA). The level of significance was accepted with *p* < 0.05.

## 4. Conclusions

Liquid SEDDS for ATR with oleic acid or Capryol 90 as oily phase, Tween 80 as surfactant and 1,2-propylene glycol as co-surfactant were developed. Based on the thermodynamic stability test, phase separation, emulsification, percentage transmittance, refractive index, turbidity, droplet size and zeta potential studies, two optimal compositions of L-SEDDS were selected—OF2 and CF3. The optimized liquid atorvastatin-SEDDS were finally successfully converted, using the spray-drying technique, to S-SEDDS. Despite the fact that liquid formulation CF3 was characterized by lower turbidity, higher percentage transmittance and better self-emulsifying properties, based on the *in vitro* dissolution study, it can be concluded that better solubilization properties were exhibited by solid formulation OF2. Obtained S-SEDDS can be formed into solid dosage forms, as multicompartment tablets or capsules, but further studies are needed.

## References

[1] Lipinski C.A. (2002). Poor aqueous solubility—An industry wide problem in ADME screening. Am. Pharm. Rev..

[2] Rao B.P., Baby B., Durgaprasad Y., Rames K., Rajarajan S., Keerthi B., Sreedhar C. (2013). Formulation and evaluation of SMEDDS with Capmul MCM for enhanced dissolution rate of valsartan. RGUHS J. Pharm. Sci..

[3] Patel A.R., Vavia P.R. (2006). Effect of hydrophilic polymer on solubilization of fenofibrate by cyclodextrin complexation. J. Incl. Phenom. Macrocycl. Chem..

[4] Vaculikova E., Placha D., Pisarcik M., Peikertova P., Dedkova K., Devinsky F., Jampilek J. (2014). Preparation of risedronate nanoparticles by solvent evaporation technique. Molecules.

[5] Karim F.T., Kalam A., Anwar R., Miah M.M., Rahman S., Islam A. (2014). Preparation and evaluation of SEDDS of simvastatin by *in vivo*, *in vitro* and *ex vivo* technique. Drug Dev. Ind. Pharm..

[6] Salimi A., Zadeh B.S.M., Hemati A., Birgani S.A. (2014). Design and evaluation of self-emulsifying drug delivery system (SEDDS) of carvedilol to improve the oral absorption. Jundishapur J. Nat. Pharm. Prod..

[7] Saritha D., Bose P., Nagaraju R. (2014). Formulation and evaluation of self-emulsifying drug delivery system (SEDDS) of ibuprofen. IJPSR.

[8] Sharma S., Bajaj H., Bhardwaj S., Sharma A.D., Singh R. (2012). Development and characterization of self-emulsifying drug delivery system of a poorly water soluble drug using natural oil. Acta Pol. Pharm..

[9] Chopade V.V., Chaudhari P.D. (2013). Development and evaluation of self-emulsifying drug delivery system for lornoxicam. IJRDPL.

[10] Seo Y.G., Kim D.W., Cho K.H., Yousaf A.M., Kim D.K., Kim J.H., Kim J.O., Yong C.S., Choi H.G. (2015). Preparation and pharmaceutical evaluation of new tacrolimus-loaded solid self-emulsifying drug delivery system. Arch. Pharm. Res..

[11] Hanh N.D., Mitrevej A., Sathirakul K., Peunqvicha P., Sinchaipanid N. (2015). Development of phyllanthin-loaded self-microemulsifying drug delivery system for oral bioavailability enhancement. Drug Dev. Ind. Pharm..

[12] Qi X., Qin J., Ma N., Chou X., Wu Z. (2014). Solid self-microemulsifying dispersible tablets of celastrol: Formulation development, characterization and bioavailability evaluation. Int. J. Pharm..

[13] Vergote G.J., Vervate C., van Driessche I., Hoste S., de Smedt S., Demeester J., Jain R.A., Ruddy S., Remon J.P. (2001). An oral controlled release matrix pellet formulation containing nanocrystalline ketoprofen. Int. J. Pharm..

[14] Czajkowska-Kośnik A., Sznitowska M., Mirkowska K. (2012). Self-emulsifying oils for ocular drug delivery. II. *In vitro* release of indomethacin and hydrocortisone. Acta Pol. Pharm..

[15] Cho W., Kim M.S., Kim J.S., Park J., Park H.J., Ch K.H., Park J.S., Hwang S.J. (2013). Optimized formulation of solid self-microemulsifying sirolimus delivery systems. Int. J. Nanomed..

[16] Trivedi K., Patel P.V. (2013). Development and characterization of liquid and solid self-emulsifying drug delivery system of fexofenadine. J. Pharm. Investig..

[17] Ravula A.R., Nagabandi V., Parney S. (2014). Encapsulation of self-emulsifying drug delivery systems (SEDDS) of lercanidipine hydrochloride into hard gelatin capsules. Int. J. Biopharm..

[18] Chen Y., Chen C., Zheng J., Chen Z., Shi Q., Liu H. (2011). Development of a solid supersaturatable self-emulsifying drug delivery system of docetaxel with improved dissolution and bioavailability. Biol. Pharm. Bull..

[19] Mahapatra A.K., Murthy P.N., Swadeep B., Prasad R. (2014). Self-emulsifying drug delivery systems (SEDDS): An update from formulation development to therapeutic strategies. Int. J. PharmTech Res..

[20] Parmar B., Patel U., Bhimani B., Sanghavi K., Patel G., Daslaniya D. (2012). SMEDDS: A dominant dosage form which improve bioavailability. Am. J. PharmTech Res..

[21] Yi T., Wan J., Xu H., Yang X. (2008). A new solid self-microemulsifying formulation prepared by spray-drying to improve the oral bioavailability of poorly water soluble drugs. Eur. J. Pharm. Biopharm..

[22] Crevar-Sakač M., Vujić Z., Brborić J., Kuntić V., Uskoković-Marković S. (2013). An improved HPLC method with the aid of a chemometric protocol: Simultaneous determination of atorvastatin and its metabolites in plasma. Molecules.

[23] Corsini A., Bellosta S., Baetta R., Fumagalli R., Paoletti R., Bernini F. (1999). New insights into the pharmacodynamics and pharmacokinetic properties of statins. Pharmacol. Ther..

[24] (2007). European Pharmacopoeia.

[25] Vaculikova E., Grunwaldova V., Kral V., Dohnal J., Jampilek J. (2012). Preparation of candesartan and atorvastatin nanoparticles by solvent evaporation. Molecules.

[26] Khan F., Islam M.S., Roni M.A., Jalil R.U. (2012). Systematic development of self-emulsifying drug delivery systems of atorvastatin with improved bioavailability potential. Sci. Pharm..

[27] Kadu P.J., Kushare S.S., Thacker D.D., Gattani S.G. (2011). Enhancement of oral bioavailability of atorvastatin calcium by self-emulsifying drug delivery systems (SEDDS). Pharm. Dev. Technol..

[28] Chouksey R., Pandey H., Jain A.K., Soni H., Saraogi G.K. (2011). Preparation and evaluation of the self-emulsifying drug delivery system containing atorvastatin HMG-CoA inhibitor. Int. J. Pharm. Pharm. Sci..

[29] Shen H.R., Zhong M.K. (2006). Preparation and evaluation of self-microemulsifying drug delivery systems (SMEDDS) containing atorvastatin. J. Pharm. Pharmacol..

[30] Talegaonkar S., Mustafa G., Akhter S., Iqbal Z.I. (2010). Design and development of oral oil-in-water nanoemulsion formulation bearing atorvastatin: *In vitro* sssessment. J. Dispers. Sci. Tech..

[31] Miryala V., Kurakula M. (2013). Self-nano emulsifying drug delivery system (SEDDS) for oral delivery of atorvastatin—Formulation and bioavailability studies. JDDT.

[32] Azeem A., Rizwan M., Ahmad F.J., Iqbal Z., Khar R.K., Aqil M., Talegaonkar S. (2009). Nanoemulsion components screening and selection: A technical note. AAPS PharmSciTech.

[33] Kibbe A.H. (2009). Handbook of Pharmaceutical Excipients.

[34] Dixit A.R., Rajput S.J., Patel S.G. (2010). Preparation and bioavailability assessment of SMEDDS containing valsartan. AAPS PharmSciTech..

[35] Patel H.K., Patel P.V., Misan C.K., Mehta D.S., Patel M.B. (2012). Development and characterization of liquid and solid self-microemulsifying drug delivery system of tacrolimus. Asian J. Pharm..

[36] Gershanik T., Benzeno S., Benita S. (1998). Interaction of a self-emulsifying lipid drug delivery system with the inverted rat intestinal mucosa as a function of droplet size and surface charge. Pharm. Res..

[37] Gupta A.K., Mishra D.K., Mahajan S.C. (2011). Preparation and *in vitro* evaluation of self emulsifying drug delivery system of antihypertensive drug valsartan. Int. J. Pharm. Life Sci..

[38] AppaRao B., Shivalingam M.R., Kishore Reddy Y.V., Sunitha N., Jyothibasu T., Shyam T. (2010). Design and evaluation of sustained release microcapsules containing diclofenac sodium. Int. J. Pharm. Biomed. Res..

